# The visualization of autism: Filming children at the Maudsley Hospital, London, 1957–8

**DOI:** 10.1177/09526951241238650

**Published:** 2024-03-31

**Authors:** Janet Harbord

**Affiliations:** Queen Mary University of London, UK

**Keywords:** autism, child psychiatry, kinesics, medical film, visual analysis

## Abstract

This article examines three films made during the 1950s by Elwyn James Anthony at the psychotic clinic for children at the Maudsley Hospital that marked an important transition in the purpose and practice of visual documentation in a clinical setting: film as a research tool was transitioning from the recording of external signs as indicators of internal subjective states, to the capture of the visual flow of communication *between* subjects. It is a shift that had a particular impact on the emergent classification of autism, a modality not yet properly separated from the broader term of psychosis, as a non-relational condition whose visual capture demonstrated a void of inter-human communicational exchange. Film was significant not only as a recording apparatus, but as a method of cutting and crafting sequences of movements into brief repetitive motifs. The filmed behaviour of children remained opaque to interpretation, a ‘finding’ that facilitated the modelling of an emergent autism as subjects who were isolated, alienated and automaton-like, inhabiting a separate temporality. The article situates this ‘second’, affectless autism, within a broader context of post-war research into gestures as a language of the body, developed largely through an intellectual network of German émigré psychoanalysts who had fled to the US and UK in the 1930s.

The role of visual culture in the history of medical and clinical accounts of psychological development is freighted by its association with empiricism. Lisa Cartwright, in her landmark study of the relationship of medicine to imaging, *Screening the Body: Tracing Medicine’s Visual Culture* (1995), identifies an historical understanding of medical imaging as the continuation of an enlightenment science of typological systems. In the work of Sander Gilman, Cartwright argues, the use of medical photography in the 19th century is regarded primarily as taxonomic, documenting anatomy and physical features of subjects in order to exhibit physical difference as an indicator of capacity and ability. Taxonomy, in this well-rehearsed argument, is the arrangement of visual features as a hierarchy of, for example, racial categories. The use of photography by Jean-Martin Charcot to demonstrate neurological affliction is but a continuation of an ideology in which the capture of surface signs on the body makes legible an internal pathology. Gilman's critique of the visual, Cartwright argues, is in accord with Jaqueline Rose's argument that the reliance on the visual as an indicator of mental pathology was only challenged when Freud questioned the meaning of the visible symptom: ‘It was only in penetrating behind the visible symptom of a disorder and asking what it was the symptom was trying to *say*’, argues Rose, that the self-evidential nature of hysteria was challenged (Rose, quoted in ibid.: 50; original emphasis). What takes place through these two critical readings, she suggests, is a revised hierarchy of the senses. Both Rose and Gilman prize speaking and listening over observing and documenting, a shift consolidated more recently with the philosopher Jean-Luc Nancy's meditation on listening that asserts its specificity over and above visuality. In Rose's account, Freud's method manifested the break from an objectifying visual regime of the clinic associated with Charcot, in turning to a cure based on the auditory.^1^

By the mid 20th century, Cartwright argues, visuality in the medical field had ‘survived its association with empiricism’, emerging in the second half of the 20th century ‘at the centre of a vast medical industry’ (Cartwright, 1995: 51). My purpose in this article is to examine a trilogy of clinical films produced during the 1950s that marked an important passage in the practice of visual documentation in this setting: clinical filming was transitioning from the recording of external signs as indicators of internal subjective states, to the capture of the visual flow of communication *between* subjects. Such an approach took departure from the psychoanalytic model of examining pathology through individual familial history, to a new focus on the here and now, the dynamic of relational interplay, or indeed its absence. It is a shift, I will argue, that had a particular impact on the emergent classification of autism, a modality not yet properly separated from the broader term of psychosis, as a non-relational condition whose visual capture demonstrated a void of intra-human communicational exchange. The filmed behaviour of autistic children remained opaque to interpretation, a ‘finding’ that fuelled conceptual modelling of diverse neurological states as isolated, alienated, and automaton-like, and inhabiting a separate temporality.

The impact of the Second World War on the psychic life of families, recently described by Michel Shapira, has produced an account of the anxiety associated with the temporary breaking of attachment bonds. Yet a more diffuse feature of war was a new fascination with covert communication; the practice of code breaking and the modelling of communication as enigmatic systems of signals focused attention on psychic relationality as both networked and disguised. ‘Scientific interest in human communication took a new turn after World War II, inspired in part by information theory and cybernetics’, David McNeill argues, a topic explored in the Macy Conferences on Cybernetics (1946–53), a philanthropically funded experiment in interdisciplinary approaches to the social, behavioural, and medical sciences. In attendance at the conferences were the anthropologists Gregory Bateson, a prominent figure of a new distinctive way of thinking about behavioural patterns across cultural, psychiatric, and environmental contexts (Bateson, 1972); and Ray Birdwhistell, the founder of kinesics, the study of body motion as an aspect of interpersonal communication (McNeill, 1992: 4). As Seth Watter has argued, the figure of Birdwhistell ‘united the most fashionable theories of social behavior, the most advanced means of analyzing behavior, the utilization of cinema to record this behavior’, establishing a method of recording and micro-gestural analysis of frames that constituted a new object of study: non-verbal communication (Watter, 2017: 35).^2^ In the field of cybernetics, the analysis of non-verbal communication patterns (relays of micro-gestures that may be in concert or in conflict with the spoken word) opened onto a new conceptualization of subjecthood as both explicitly and covertly, consciously and unconsciously, relational. In the discipline of psychiatry, the question of a body's intelligibility emerged as a key indicator of ‘pathological’ states. Whether a patient's movements could be mapped onto a matrix of shared code, preferably exhibiting intent to communicate, held particular significance for clinical research of conditions within which spoken language was not present, such as an emergent autism.^3^

Film was particularly valuable as a clinical research tool with a capacity to record behaviour as patterns of movement, orientation, and reaction. During the 1950s, the act of recording (or documentation) became eclipsed by methods of visual analysis afforded by post-production, where meaning could be constructed through the assembly of film materials according to different variables. Clinical films could be assembled along the principle of self-injury, facial tics, or comportment, providing a database of behaviours rather than narratives of individual or generic development (for example, James Robertson's films of infants). This article examines three films made under the direction of psychoanalyst Elwyn James Anthony at the Maudsley Hospital during the years 1957–8 that, as a series, track the changes in approach to and conceptualization of ‘psychotic states’, facilitating an emergent definition of autism in the decade that follows. These little-known yet particularly data-rich illustrations of clinical research, according to Bonnie Evans, play a significant part in the history of autism in Britain (Evans, 2017, 2019, 2024), while I have argued that these films contribute to the division of bodily gesture into typical and atypical formulations (Harbord, 2019, 2021: Eastwood *et al.*, 2022). The first film, *Approaches to Objects by Psychotic Children* (1957a), recorded with a static camera in a small room, documents children undergoing a series of tests. The second, *Aspects of Childhood Psychosis* (AOCP; 1957b), is conceptually and formally the most complex of the three, presenting short sequences of children in motion, their movement cross-referred to diagnostic categories. The third film, *The Natural History of a Psychotic Illness in Childhood* (1958b), is a case study of one child, Margaret, where the standard markers of documentary narrative and form shape the enquiry as an unfolding story of developmental disorder. As a trilogy, the films bridge a behavioural approach to clinical work characteristic of the previous decade, and a documentary style of empathetic observation of the ‘patient’ prefiguring the educational films of the decade to come.

The term *psychosis*, which appears in each film title, was used interchangeably during this decade with a number of others with whom its relationship is not clearly defined. ‘Unlike Kanner's more or less coherent syndrome of “early infantile autism” (1943)’, writes Berend Verhoeff, ‘diagnostic terms such as childhood psychosis, childhood schizophrenia, “atypical child”, and autism were used rather loosely and interchangeably in a psychoanalytical approach to infantile psychopathy and problems with developing relationships’ (Verhoeff, 2016: 119). Autism featured more often in clinical work as an adjective – as in the description of ‘autistic withdrawal’, for example – rather than as a noun, although this was about to change. In a detailed history of autism in Britain, Bonnie Evans argues that the meaning of the term *autism* undergoes a reversal in the post-war period, in which Bleuler's description of autistic states of excessive infantile hallucination and fantasy is turned inside out: autism moves from hot to cold, becoming a model of affect and fantasy deficiency (Bleuler, 1911). ‘From the mid-1960s onwards, child psychologists in Britain used the word “autism” to describe the *exact opposite* of what it had meant up until that time’, she states (Evans, 2017: 10; original emphasis), a critical shift aligned with a broader transition in the way that mental illness was conceived within the post-war period that Katie Joice identifies as ‘no longer characterised by an excess of emotion but by affectlessness’ (Joice, 2020: 109).

This article examines how the three films produced at the Maudsley contributed to the transformation of the meaning of the term *autism* during the 1950s, preparing the ground for the emergence of a ‘second’ autism in the following decade. While the films exercise different approaches to the subject, each presents gestures as symptoms that could, in the editing room, be cut from the flow of activity, and as isolated features, presented as illegible hieroglyphs, ambiguous signs from another world or time. With the foregrounding of bodily movement, clinical attention, which until this point had tended to focus on autistic verbal atypicalities such as echolalia, became inclusive of questions of the intelligibility of gestures. The films demonstrate what Israel Kolvin, in his follow-up study of Anthony's work, published in 1971, would select as a key feature of early-onset autism, in ‘stereotyped movements’ and ‘poor relations’. Evans argues that it is through Israel's testing of Anthony's hypothesis that the second autism, drawn from a model of early-onset psychosis, ‘became dominant’ and its features properly mapped (Evans, 2017: 237). That is not to say that film revealed autism, or that autism was diagnosed as a result of certain film techniques, but that film and autism are constitutive elements in the same field of events in which the body's intelligibility, its capacity to communicate and be read, was undergoing a thorough remaking.

## Filming at the Maudsley

In 1948, in a bid to adjust the reputation of the Maudsley Hospital Medical School, its name was changed to the Institute of Psychiatry. According to historians Edgar Jones and Shahina Rahman, if the remit of the Maudsley had in part been to raise the status of academic psychiatry in Britain, in its early years it had struggled to establish itself as a respected research institution. It was only shortly before the Second World War that it began to gain traction in this respect with approval of research funding awarded by the Rockefeller Foundation (Jones and Rahman, 2009). The work of Elwyn James Anthony and Kenneth Cameron in the psychotic clinic for children was part of a drive for the Maudsley's reputational gain, feeding into national legal frameworks legislating for ‘maladjusted children’ (Evans, 2017). The deployment of film within the clinic spoke to the interplay of these various interests and positions. Film provided documentation of the research being conducted in the children's unit, and with findings circulated internationally, the method promoted the Maudsley's institutional reputation. Furthermore, the capacity of film to record movements that could be extracted from the context as though they were discrete units appeared to offer a method for greater clinical precision in the segregation of so-called disease entities.

In 1952, Anthony was appointed as Senior Consultant to Children at the Maudsley Institute (Anthony, 2010). His task, as he describes it in a lecture to the British Psychological Society in April 1958, was ‘the reinstatement of the psychotic within the theoretical framework of our normal practice’ (Anthony, 1958a: 215).^4^ As a clinician, Anthony was exposed to a wide range of eclectic influences, beginning with an upbringing and training fundamentally shaped by the forces of the British Empire, and subsequently the Second World War. Born in Calcutta, India, in 1916, and educated by Jesuits in Darjeeling, Anthony emigrated to Britain to train in medicine at Kings College.^5^ During the war, as a serving officer, he was assigned a posting at Hollymoor Military Hospital, Northfield, where he met Sigmund Foulkes, the psychoanalyst with whom he would collaborate on a book that was to establish the foundations of group psychotherapy. Foulkes had trained as an analyst in Frankfurt in the sociopolitical milieu of the Frankfurt School of Marxist sociologists, encountering Max Horkheimer, Herbert Marcuse, and Frieda Fromm-Reichmann (Harrison and Clarke, 1992). At Northfield Military Hospital, Foulkes led a second series of therapeutic experiments between 1942 and 1948, building on the work of Wilfred Bion and John Rickman in the first series, utilizing the therapeutic properties of groups.^6^ The experiments in group treatments provided an economy to dealing with the number of war-traumatized patients; it also represented a British iteration of a psychoanalytic and psychiatric turn towards relationality that was concurrently being debated in the US. Northfield was, according to Sidney Bloch, a centre of innovation where Bion and Foulkes experimented with group methods of treatment that were to prove influential in the following decades. Bloch writes, ‘After the war Bion's work influenced therapists of the Tavistock School while Foulkes was the all-important figure behind the establishment of the Institute of Group Analysis’ (Bloch, 1986: 741). The Northfield experiments symbolized the social and political importance of psychiatry, both during the war and in its immediate aftermath, in its treatment of war trauma, and yet, write Harrison and Clarke, ‘important lessons were learned and subsequently forgotten’, arguing that those practising therapeutic group analysis in the health service were continuously obliged to defend its methods (1992: 698).

Following the war, Anthony applied to train at the British Psychoanalytic Institute, and was allocated Foulkes as his analyst. In an account in which he reflects personally and professionally on his relationship with Foulkes, Anthony recalls Foulkes as figure who maintained ‘a steely mastery of technique’ in a manner that could appear dismissive and remote. ‘I … pulled out my history of a very disturbed childhood conducted, without the help of parents, high up in the Himalayan Mountains and tutored by Jesuit priests’, he writes, in the face of which Foulkes appeared unmoved. Yet the analysis with Foulkes was formative, leading Anthony to reflect during a year's training with Jean Piaget, in Geneva, that ‘I became more intensely aware of my dedication to the understanding of children’ (2010: 83). Towards the end of his analysis, Foulkes invited Anthony to attend group meetings at his home, where ‘I found myself surrounded by psychiatrists, sociologists, developmentalists, philosophers, psychologists and a range of academicians all immersed in an extraordinary interchange of a huge variety of opinions and reactions’, and ‘it began to move me from my psychoanalytic position into a group-analytic world that seemed to be in the making’ (ibid.: 82–3). The figure of Foulkes, with whom Anthony worked closely during this decade, is a critical connector or conduit between these various constituents of research whose interests were the body's communicative capacities, therapeutic treatments, and experimentation in techniques of observational research.

The three films that were made at the Maudsley each take a different approach: a documentation of clinical testing, a clinical taxonomy of psychiatric conditions, and a narrative account of autism as it emerged in a single child. The first film, *Approaches to Objects by Psychotic Children* (1957a), observes children undertaking object permanence tests. Filmed on 16 mm black-and-white stock in a small hospital room, a fixed camera records the activities of each child, seated at a table, as they dutifully respond to questions and tasks, presented by a person (left of field) whose arm is the only visible marker of their presence. Far more imposing on the scene is the apparatus of recording, evident via the children's preoccupation with the camera, the operator, and the strong film lights: the children smile into the camera or just above it, look away and back again, stare distractedly at the lens, and squint into the harsh light. The clinical framework marking this event as research appears in the form of a letter/number allocated to each child for each test, situated on the wall in the back right of the frame. Intertitle cards explain the test in a summary of Piaget's theory of object permanence, a set of stages through which a child moves from an early age, initially demonstrating a disregard for the object through to an understanding that when out of sight it continues to exist. The use of film is straightforwardly to document the procedure of clinical testing, to extend the opportunity, as it were, for the observation of clinical practice. Notably, the intertitles make no mention of the formal limitation of the absence of sound recording, nor is there reflection on the demonstrable effect that the recording apparatus has on the experience of the children during testing.

The film AOCP is a more complex, contradictory, and powerful presentation of clinical work whose post-production techniques bind film form to clinical content. That is, the extraction of children's gestures from the context of their recording, and their recombination as data sets, transforms sequences of activity into brief repetitious acts. These repetitive and ‘restrictive’ behaviours were to become a key visual marker of autism in its future classification (Wing, 1981), ‘the motions that are most typically read or made legible on the autistic body’ (Yergeau, 2018). A research film that observes children at play, AOCP is notable for its minimal narrative, its lack of sound, and, in the opening sections, the use of intertitles directing the viewer to read for certain gestures as symptoms. In total, almost 30 minutes of black-and-white footage discloses the interior of a clinic populated by children between three and nine years of age, engaged in various activities: in the presence of nurses and doctors, children play with sand, flick a light switch on and off, grab at a milk bottle, hop on the spot, place a blanket over their head. Children sit on laps and stare into the lens, glance back and forth between the camera and its operator, and squint into the bright film lights. The film-making apparatus, that is, visibly operates as a presence in the scene being observed. This is a film that Anthony refers to as ‘giving expression to my belief in the continuity of psychosis with the other diagnoses of child psychiatry’ (Anthony, 1958a: 214).^7^ The film's front-loading of information in the form of intertitles privileges the instructional over the evidential in the first part of the film; intertitles constitute approximately a ratio of one-third (88 sequences) to two-thirds recorded sequences of filmed children (164), with 66 intertitles appearing in the first 10 minutes of the film. As the volume of intertitles decreases, the film moves into a mode of short sequences of children at play. It is likely the film was shot on a spring-wound camera with a shot length limited to approximately 20 seconds, placing a formal limitation on the material, but in the editing room these sequences were reduced to far shorter sequences of between 4 and 8 seconds.

The opening and closing sequences are notably distinct from the main content of the film. As ‘bookends’, they personalize the data that the film delivers through a series of photographs of children shown first as babies and then several years later, by which time their faces, according to the title cards, register ‘signs’ of psychosis. The film closes on an example of a boy who is singled out for his capacity to learn to engage: the sequence begins with a montage of interactions between the boy and a doctor within the clinic, culminating in a shot filmed in the grounds of the hospital in which the boy runs into the arms of the same doctor. Yet between these two more affective fixtures, the film's most pronounced formal properties are short clusters of fragments of ‘behavior’, decontextualized recordings of movement. A cinemetric analysis of AOCP, an approach from film studies that sifts the film's underlying construction through decisions of cutting and joining sequences in post-production, shows the average shot length of the film to be 7.5 seconds.^8^ In the camera's close attention to the children in the foreground, the viewer is invited to follow their activities against an inert background populated by the shadowy figures of nurses and doctors, unlit and barely discernable, their heads and torsos regularly cropped from the frame of reference. Nonetheless, they are the functional adjuncts to what is being captured on film, which is the refusal of relationship. Multiple scenes begin with an outstretched hand in the frame, which becomes more persistent in its offering of objects ranging from toys and individually wrapped sweets to bottles of milk, or a hand reaching in to retrieve an object, such as a book. The camera observes the reactions of children, their facial expressions, and the ways in which their bodies move in response to both things and people, yet it is almost exclusively the impossibility of the intra-human relation that is presented.

The brief sequences of filming bear a resemblance to what Katie Joice has described as a privileging of the ‘temporal fragment’ in post-war social science, a recorded detail that is removed from the flow of time and place, and subjected to intense scrutiny (Joice, 2020: 110). The practice of microanalysis, she writes, not only brings into view ‘small behaviours’ that otherwise evade the eye, but (following Stephen Kern) reconfigure space and time, particularly in increasing the density of lived experience as a ‘thickened reality’ (Kern, 2003: 81). Cinema, as a time-based medium with the capacity to compress, extend, slow, accelerate, and create ellipses, inevitably shapes an experience of temporality (Charney, 1998; Doane, 2002). The temporality of AOCP, however, achieves the opposite of microanalysis: the clipped gesture, a fragment of a longer sequence, isolates the body's animation. The film's method is extractive: movement is lifted from its environment, a radical decontextualization that emphasizes and energizes activity. Removed from the wider context, the children's actions become a compendium of rocking, shaking, flicking, grimacing, hopping, and jumping, prefiguring a contemporary fascination with decontextualized images of bodies in movement set on loops of pleasurable repetition (images in Graphics Interchange Format, or GIFs). The children as they jump, flap, and grimace, appear to be figures caricatured by their own automatism. As if to underscore this association, Anthony includes in the film one drawing by a child: it is of a human–robot hybrid figure.

A second consequence of the editing techniques is that the formal non-relational quality of the shots amplifies the non-relational features of the emotional environment. Unlike the classical use of cutting and joining film in fiction to build suspense or to create a narrative arc, the arrangement of shots does not follow a logic of suspense and active questioning (if A follows B, what then of C?), but a logic of (non-relational) addition. The brief shots ‘stacked’ one on top of the other suggest that they are linked by an affinity (A is like B is like C), within which qualitative variation is found. The brief sequences are not dialogically engaged in the production of a meaning that unfolds through points and counterpoints, but singular instances of a behaviour, the film's structure subtending the diagnosis that these children are not ‘in relation’. The commentary of the intertitle cards offers the terms of reference for autism, caught here in the process of sedimentation as a clinical profile: ‘rigidity of behavior and endless repetition’, ‘flat affect’, ‘bizarre motor behavior’, ‘robot-like drawings’, ‘primitive relations with things’. Fixed in their gestures of rigidity and repetition, the children's refusal of hands and their busy preoccupation with objects and materials translates as ‘devitalising people into tools’.

In contrast to Anthony's first film of children undergoing tests, AOCP had an identifiable occasion, purpose, and addressee, the typical features of institutionally produced, or ‘useful’, films according to media historians Vinzenz Hediger and Patrick Vonderau (2009: 10). AOCP was Anthony's presentation of the most significant Maudsley research to the Second International Congress of Psychiatry in Switzerland, a large international gathering of experts in the field of psychiatry: 1900 participants from 59 countries were registered (Campbell, 1958: 318). In September 1957, Anthony travelled to Zurich to present the film at this event, whose subject was ‘The Present State of Our Knowledge About the Group of Schizophrenias’: contributions addressed the cause, diagnosis, aetiology, and symptomatology of this umbrella term that was regularly invoked alongside psychosis. Published reports of the conference, however, are somewhat disappointing, with a headline in an American journal claiming that ‘nothing startlingly new’ was presented (ibid.). One of the attendees at the conference was Leo Kanner, who delivered an account of his current work. In his report of the event published the following year, Kanner failed to mention Anthony's film (Kanner and Eisenberg, 1959: 609). Seven months after the Geneva congress, Anthony delivered a public lecture in which he expressed a degree of scepticism towards the psychiatric community and the ‘cult’ of naming psychiatric conditions after ‘distinguished pioneering clinicians’. Noting wryly that ‘there did not seem to be a sufficiency of symptoms to share out among the various prospectors’, Anthony singles out Kanner for the particular criticism of vigorously resisting any ‘territorial encroachment’ upon the term *autism* (Anthony, 1958a: 213). This perhaps was a retort for his film, and research more widely, not having been given sufficient recognition: in AOCP, Anthony had included a roll call of psychiatric luminaries including Margaret Mahler, Michael Heller, Lauretta Bender, and indeed Leo Kanner.^9^

What might Anthony have aimed to achieve with this film, and what was the outcome in clinical terms? In a written reflection on the research conducted at the Maudsley, including this film, Anthony states his aim as ‘attacking a conception of childhood psychosis as a bizarre, atavistic condition’ (Anthony, 1958a: 211). ‘Every child begins its life in an autistic state’, he writes, emerging from this state when the environment offers a constancy of stimulation (ibid.: 216). Autism as a developmental stage persists when an environment fails to provide adequate stimulation (in the provision of care), leading to ‘non-emergence’. Or, when a child is overstimulated, autism becomes a form of retreat, a model that Anthony links to Freud's concept of an infant's ‘protective barrier’ in *The Pleasure Principle* (1922), and to the sensitive children with an inadequately ‘thin’ protective barrier proposed by Bergman and Escalona (1949). The instances of primary and secondary autism described by Anthony manifest constitutional barriers that are either too thick, in the case of the former, or too thin, in the case of the latter (Anthony, 1958a: 218). In either regard, the concept of a constitutional barrier that is ineffective brings into view a world of overwhelming sensory stimulation that a subject must establish defences against, such as creating routines or rituals to impose order. In this, autism might be positioned historically on the same horizon as Jonathan Crary's late 19th-century modern subject, whose capacity to protect against the excessive sensory stimuli of an increasingly technologized and mediated urban environment was only ever partially achieved (Crary, 1999: 362).

If AOCP was Anthony's professional bid for recognition from a clinical community, the third film, *The Natural History of a Psychotic Illness in Childhood*, appears to have different aspirations: to address a non-specialist audience, adopting the mantle of public information films to come in the following decade that would seek to educate the audience through individualization and domestication of psychiatric conditions. The focus of the film is an individual child, Margaret, whose condition is given narrative form by the film and socially contextualised in the broadest sense. Spatially, the story has broadened to include locations outside of the clinic, locating psychosis in familiar milieu: a street, a playground, Margaret's home and garden, countryside, the seaside. Structurally, the film is a story recounted by a narrator. The film opens with an arresting image of a group of children in an urban space, walking authoritatively and directly towards the camera in a manner that immediately breaks the fourth wall of cinema. The sequence records apparently ‘normally’ developing children in an urban milieu, yet statistically a high percentage of children will require ‘psychiatric guidance’, and very occasionally, the narrator announces dramatically, a child will develop psychosis. The film visually produces this difference in a striking sequence where Margaret is introduced. A long shot records Margaret walking towards the camera, holding the arm of a nurse: the nurse looks at Margaret while Margaret looks at the ground. The next sequence films Margaret in close-up sitting in an armchair in a field of long grass. It is a surreal introduction, a bucolic setting in which the domestic feature of the comfortable armchair is incongruent and visually jarring. This introduction of Margaret effectively marks her ‘pathology’ through its *mise en scène*, mixing the signifiers of domestic and public space, internal and external worlds.

The story of Margaret is the story of a pathology unfolding over time, drawing on three technologies of inscription, each of them documents from the domestic sphere: family photographs, home movies, and a mother's diary.^10^ Photography perhaps was seen to contribute something more personal as well as evidential to the cases described.^11^ The photograph album in this film positions Margaret within the timeline of a family, and like Kanner's case histories, describes the occupations, sensibility, and comportment of the parents. It also moves back in time generationally to explore the possibility of hereditary conditions. *The Natural History of a Case of Psychosis in Childhood* prefigures what is to come in the following decade as autism as a diagnostic category begins to stabilize as separate from psychosis, and autistic children become the topic of a rising number of documentary films addressed to a general public. In the UK, *Illustrations of Childhood Autism* (Dermod McCarthy and Harold Lowenstein, 1969), shot in Stoke Mandeville Hospital, would recount in marked sympathetic tone the plight of an autistic child named Lucy, using the practice of stop-motion to pause on the smallest gestures, ‘the outward sign of bizarre thoughts and fears’. In the US, a film titled *Autism's Lonely Children* is broadcast on the National Educational Television Network in 1964, a pilot study of twin autistic boys, Marty and Peter, by Frank Hewett of the Neuropsychiatric School at UCLA. The film opens with the remark that ‘time stands still’ for these children who inhabit a separate world where a shared temporality does not apply. In a similar hybrid of educational-emotive appeal, a American documentary about an autistic four-year-old girl, *A Time for Georgia* (Peter Scheer, sponsored by the Pre-schoolers Workshop, 1970), is screened, winning an award.^12^

The many temporal references in these films about autistic children to an atomized and etiolated sense of time that fails to register change or to structure experience recall a modernist cultural aesthetic. Patrick McDonagh argues that the very same features through which autism was to become diagnosed and defined – ‘isolation and alienation, the need to establish personal rituals to impose order on the world, the removal of referential and conventionally communicative functions from language – also appeared as critical components of the modern (and modernist) identity’ (McDonagh, 2007: 113). The case for an alliance or series of correspondences between autism and modernist literature has been made by a number of prominent scholars. Ato Quayson, for example, argues that the problems of the interpretation of language that autism historically presents are mirrored in Samuel Beckett's novel *Murphy*, where the author ‘institutes a series of gaps between language and its discursive referents … an epistemological impasse between narrative and the representation of emotion’ (Quayson, 2010: 838). Julia Mieles Rodas notes the reverence for autistic features or affinities in the realm of art and literature, and their denigration in the realm of psychiatry (2018). Sonja Boos identifies in Kafka's fictions unintelligible gestures that ‘shift our focus to the concrete manifestations of a very real aspect of modernity: the way in which the body becomes the playing field upon which medical science and the humanities will increasingly pursue their interests’ (Boos, 2019: 845). One might also make the case that modernist affordances of film, particularly the practices of montage in post-production, produced an aesthetic that separates events and atomizes subjects; attends to surfaces, patterns, and planes; and remains on the outside of gestural intelligibility.^13^ McDonagh makes the argument, through literature, that ‘modernity and modernism made possible the recognition of autism’ (2007: 101). I would make a similar argument for the import of film in the recognition of autism as a category. As a constituent part of the milieu in which debates about the mind, capacity, and relationality pivoted on an understanding of the body in motion, film was able to capture, demonstrate, and model the intelligibility, or otherwise, of bodies.

## Denaturalizing histories of communication

If, in the mid 20th century, an emerging picture of autism as a condition of indecipherable gestures, social isolation, and affectlessness was gradually cohering, it was against a background of research into typical communicative ‘behaviour’ that the contours of autism were brought into relief. During the 1950s, a decade in which the communicative capacity of the human body was subject to intense speculation and enquiry, the visual ‘conundrum’ of autistic body movements posed the question of whether autism had a body language at all. As Brenda Farnell argues, anthropological interest in visual aspects of human communication and movement considerably predates a new form of public-facing research emerging in the 1950s and 1960s that used filmed data as their primary material (Farnell, 2003). Building on the work of anthropologist Franz Boas and linguist Edward Sapir, who during the 1920s had demonstrated the significance of non-verbal behaviour in communicative acts, a range of interdisciplinary scholarship sought to understand the dynamically embodied action scripts of social encounter. George Trager and Edward Hall, working at the Foreign Service Institute in the US Department of State, had in 1953 published their work *The Analysis of Culture*, a study of communication behaviours external to spoken language (Hall and Trager, 1953). Hall was to go on to develop the subfield of proxemics, a topography of spatial features of communication practice, simultaneous to Erving Goffman's sociological modelling of a dramaturgical presentation of a self that was mobilized situationally (Goffman, 1959[1956]). However, the most famous theoretician of bodily communication, whose work crossed over into the public realm and captured imagination with the term ‘body language’ (a term that he later came to regard as a vulgarization of his method; Watter, 2021: 250), was the anthropologist Ray Birdwhistell.^14^

The bold premise of Birdwhistell's thesis was that at least three-quarters of communication in any social exchange was non-verbal, and film was a crucial tool sensitive to minute corporeal flexes and tones (Birdwhistell, 1952). Birdwhistell recorded social interactions on 16 mm film, projecting the recordings at varied rates to reveal micro-gestures that challenged the primacy of verbal exchange. This time-consuming method of frame-by-frame analysis is a practice that Martha Davis describes as the use of ‘film projectors as microscopes’, a blend of scientific and aesthetic instruments (Davis, 2001–2).^15^ Farnell contends that without a conceptual model to make bodily movement finite, and therefore suitable for analytical purposes, ‘Birdwhistell's analyses tended to dissolve into microanalytical minutiae from which he seemed unable to emerge’ (Farnell, 2003: 50). Yet the expanding field of human interaction studies was able to demonstrate the intellectual gains of reading the body as a semiotic text through exemplary detail, from the value of studying eye movements during an exchange, to the meaning of how proximate a speaker chose to be to others. The broad strokes of this work delivered a concept of interaction that included conscious and unconscious acts, verbal and non-verbal ‘behaviour’, that was orchestrated in and through the body. Positioned against this expanding definition of how and when individuals were in correspondence, autism appeared as the limit case, an extreme instance of non-complexity in the field of human communication studies.

The recordings of children at the Maudsley demonstrated such ‘failures’ of communication, represented in vignettes of a refusal of contact and instances of physical and social isolation. Anthony was not unaware of the extensive research in the field of communication; indeed, his work with Sigmund Foulkes directly engaged with some of the same ideas in the study of group dynamics in a psychoanalytic context. Since meeting in 1941 on the tennis courts at Northfield Military Hospital, Anthony's relationship with Foulkes had evolved on many fronts. In 1952, Anthony and Foulkes were part of a group of experts from cognate disciplines, including the German sociologist Norbert Elias, to establish the Group Analytic Society in London. In 1952, Foulkes had attempted, unsuccessfully, to establish an NHS unit for group psychotherapy at the Maudsley Hospital (where he was consultant physician from 1950 to 1963).^16^ In the same year that Anthony presented the film AOCP to a conference in Zurich, Anthony and Foulkes published the co-authored work *Group Psychotherapy: The Psychoanalytic Approach*, a book that followed on the heels of Foulkes’ earlier inaugural text on group therapy *Introduction to Group Analytic Psychotherapy* (1948). The co-authoring of the book Anthony describes as ‘a new development in our relationship’, one in which Anthony appeared to have become a more equal partner to Foulkes, a promotion from his status as junior analysand. ‘We worked separately, and quite independently to produce a volume that became an immediate best seller’, Anthony recounts in a faintly romantic mode, ‘and our two names seemed to be forever linked together’ (Anthony, 2010: 83).

Marking the occasion of Foulkes’ death in 1976, Anthony's obituary recalls this decade as a rich braiding of influences; he was not only Senior Consultant to Children at the Maudsley, where he worked with Kenneth Cameron, and part of the emerging movement of group analysis in Britain with Foulkes, but also a lecturer at the Anna Freud Institute (Anthony, 2010).^17^ In contrast to a well-rehearsed historical account of the mid-century development of psychiatry and psychoanalysis in Britain, which foregrounds the dramatic moment of the Freud–Klein controversies of 1941–5 (King and Steiner, 1991), Anthony's career brings into view the 1950s as a decade characterized by a more dynamic, international, and fluid set of debates in which child psychiatry was being shaped by interconnected and interdisciplinary enquiries about human communication, embodiment, and capacity. Foulkes was a key connection for Anthony during this time. When Foulkes had fled Germany following the rise of Hitler as chancellor in 1933, and arrived in London, via Switzerland and France, he had taken his departure from a group of radical analysts who were forced, like him, to become what Paul Weindling names ‘alien psychiatrists’, scattered across Europe and the United States (Weindling, 2010). Foulkes, along with Frieda Fromm-Reichmann, had trained at the Neurological Institute in Frankfurt and worked with the dynamic psychiatrist and neurologist Kurt Goldstein. Foulkes and Fromm-Reichmann shared a political sense of the powerful potential of psychiatric treatment to create transformation beyond the individual, to affect the complex interrelationships of groups or systems, be that families, cultures, or societies. Fromm-Reichmann fled to Strasbourg and then Palestine, settling eventually in the United States. Shortly after her arrival, she became a resident psychiatrist at Chestnut Lodge Hospital in Rockville, Maryland, the institution that Anthony would, towards the end of his career and having also taken up permanent residency in the United States, become director. In the years in which Anthony and Foulkes were co-authoring their book on group analysis (1955–6), Frieda Fromm-Reichmann was initiating what was to become the most famous experiment of this decade in the field of social communication, a research project that was methodologically dependent on film: the Natural History of an Interview (NHI).

In the detailed account of the NHI by Wendy Leeds-Hurwitz and Adam Kendon, the motivation for the project arose with Fromm-Reichmann's ambition to understand the precise moments in the psychoanalytic encounter when a patient is most receptive to understanding and insight (Leeds-Hurwitz and Kendon, 2021: 148). She was awarded a fellowship at the Centre for Advanced Studies in the Behavioral Sciences (CASBS) in Palo Alto, to further her ambition to identify moments of treatment success in order to improve her method and better inform her teaching. Initially, Fromm-Reichmann recruited the linguist, Norman McQuown, to study transcripts of her sessions with patients at Chestnut Lodge, subsequently involving a number of other colleagues (Henry Brosin, expert in interpersonal psychiatry, and Charles Hockett, whose expertise was descriptive linguistics) at the CASBS. With the realization that the body movements of patients were as significant to the process of therapeutic work as spoken words, Fromm-Reichmann expanded the group further, issuing invitations to anthropologists Ray Birdwhistell and Gregory Bateson, the latter additionally an expert in information theory with extensive experience of using sound-synchronous film in his research into schizophrenia and interaction. With the inclusion of Bateson, the assembled researchers decided on the analysis of a series of three films that Bateson had recorded with a participant known in her anonymised name as Doris: the films were recorded in her home during July 1956. Although Doris was undergoing psychotherapy, the focus of research had shifted from a clinical context to a domestic setting. The materials were subject to fine-grained analysis through a process known as ‘soaking’, the repeated viewing of the films leading to a selection of key sequences for detailed analysis and transcription. The project, initiated conceptually in 1955, continued in some form until 1968. The team worked together on the materials for a year, and then subsequently in disparate institutions, for a further 10 years or more, without the findings ever being published as a complete account. The NHI, however, is frequently credited for its innovations, providing an influential new model of communication research in a ‘natural’ setting, for the ‘first systematic analyses of both verbal and non-verbal aspects of social interaction’ (McElvenny and Ploder, 2021: xiii), and the use of continuous visual recording as a primary tool.^18^

Henning Engelke suggests, drawing on Adam Kendon's assessment of the NHI, that the project marks a change in the conceptualization of communication in this decade, moving away from an understanding of gestures as symptomatic of inner states and towards a model of individuals operating within systems of relay, and in film embedded within an environment. ‘The project initiated a shift from film as a means of capturing individual expression to a perspective, informed by cybernetics and systems theory, and based on filmic “specimens”’, he writes, ‘that regarded the actors in human communication “as participants in complex systems of behavioral relationships instead of as isolated senders and receivers of discrete messages”’ (Engelke, 2021: 109, citing Kendon, 1979). If kinesics expanded the semiotic field of meaning to include manifold movements of the body, systems theory introduced the idea of feedback loops, continuous communication wherein meaning is incrementally and rhythmically produced between participants: that is, meaning is immanent to the participants and emergent in the situation. For Katie Joice, Birdwhistell's method was complicit in the identification of maternal affectlessness as a pathological factor in an infant's development, an instance wherein the communication loop is incomplete (Joice, 2020). Mothers, she argues, ‘particularly emotionally absent mothers, formed the ontological, and social, backdrop to his research’, a foundation upon which a model dividing healthy from pathological communication could be situated (ibid.: 110).

What, then, are the implications of this research, and its mapping of bodily communication, for the films made concurrently at the Maudsley, and the diagnosis of autism? On a purely comparative level, Anthony's films appear to evidence a communicative deficit in a group of children existing outside of the complex communicative relay apparent in the interactions recorded in the NHI. However, the kinetic study of behaviour did not simply support typical accounts of communication, but challenged a number of normative precepts, most significant of which (in the context of this discussion) is intention (Yergeau, 2018).^19^ The historical valorization of intention as a driver of social communication-as-expression was radically downgraded by the assertion that communication was not necessarily, and in Birdwhistell's view only minimally, a conscious act. The implication of kinesis, borne out by the films of the NHI, was that nobody was fully in control of their gestures, that signification was somehow in excess of conscious intent. Indeed, it was quite probable that an individual's spoken account was at variance with their bodily communications. By reducing the value of intention in a communicative event, this insight made the question of what was meaningful behaviour more difficult to identify, leading to methodological problems in defining the object, or indeed field, of study. In a sense, the NHI was suggestive of communicative complexity in all contexts, including the clinic. What is conclusive about the difference between these two types of research film that are each attentive to body language, yet in different ways, is that the language of film – the choice of film techniques in the stages of recording and post-production – is implicated in determining the outcome. The sense of a thickened, dense time that accompanies the NHI materials is produced through long takes: in film historical terms, a Bazanian preference for duration within a research context. In contrast, the fragmented, incoherent time of the clinic arises from a style of editing that might be more closely aligned with Eisenstein's fractured montage of a clinical scenario, capturing the titular ‘aspects’ of an environment as colliding shards.

The echo of the NHI in the title of *The Natural History of a Case of Psychosis in Childhood* suggests that an emerging multidisciplinary approach in anthropology, linguistics, and communication studies, appropriating from natural history the method of observing an unfolding process, was in the ether. If Anthony was at all influenced by the method of observational recording of interactions, he was not persuaded that the medium of film was critical to this enterprise. The three films he made at the Maudsley appear to be his last. They also mark the end of his career at that institution, and indeed in Britain. In the year that he completed the film of Margaret (1958b), Anthony accepted a post in the United States, taking up the prestigious position of the first endowed chair in child psychiatry, the Blanche F. Ittleson Professorship, at Washington University. He continued to research in the field of child development, conducting longitudinal studies of children and producing influential publications and guidance on the concepts of resilience and risk in childhood, the topics for which he is mostly remembered (Anthony, 1974; Anthony and Cohler, 1987).

The trilogy of films made at the Maudsley offer nothing conclusive to the identification of autism and its causes in the post-war period. Anthony's reflection on the outcomes of this period of experimental research as ‘partial knowledge’ received in ‘our current state of ignorance’ indicates the degree to which he is reluctant to put forward a specific claim (1958a: 211). The films, rather, are an attempt at a comprehensive mapping and visual illustration of theories of childhood psychosis in the late 1950s, with autism emerging as a distinct category apart from psychosis. This work proved influential to those who followed, among them Michael Rutter, as experimental research transitioned into a more distinct field of autism research. In 1966, Rutter was appointed consultant psychiatrist to the Maudsley, and two years later he published an article in the *Journal of Child Psychology and Psychiatry* that continued Anthony's efforts at mapping the field of experimentation and analysis: ‘Concepts of Autism: A Review of Research’. In a manner that borrows from Anthony's scoping of theoretical positions, Rutter arrived at a tentative conclusion: that autism was not the result of styles of parenting or social deprivation, nor a form of childhood schizophrenia, but the result of a cognitive difference that manifest in language and perception. ‘Of all the hypotheses concerning the nature of autism’, he writes, ‘that which places the primary defect in terms of a language or coding problem appears most promising’ (Rutter, 1968: 21). What is striking in Rutter's summary is the echo of computational language, the imaging of language ‘defect’ as a type of coding problem, leading to a lack of relay or looping, a view consonant with the visual presentation of autism in Anthony's films.

The films did not deliver an outcome in terms of a nosology of autism. Rather, they were part of something of a different order: an increased attention to the intelligibility of bodies-in-motion that was playing out variously in the domains of social science, anthropology, and psychiatry, which laid the foundations for the classification of autism as a communication deficit. Film was not simply a vector transmitting communication effects, but an apparatus whose techniques of documentation and post-production atomized and automatised the subjects recorded. However, viewed through the lens of 21st-century interests, the films evidence something else in the behaviour of these children: a deep engagement with the material world of things and a sensitivity to the sensory aspects of the environment. The recordings of children at play reveal a fascinated interest in textures, patterns, and material properties of things, a fascination that gave rise to interactions of rhythmic intensity, or trance-like states of reverie. In other words, an arresting feature of the films is the way in which these children have not succumbed to the demotion of the material world as a feature secondary to human significance and signification systems. Accompanying this contemporary insight, the films afford something equally stark: the visualization of clinical speculation itself. The films implicitly capture what the historian of science Hannah Landecker has described (of a different moment and context) as ‘scientific looking’ itself (Landecker, 2006: 121). A repeated visual motif in the recorded sequences is the quizzical look into camera as the children try to puzzle what it is that is looking at them. Under such scrutiny, autism in 1957–8, not quite a category of its own yet, is becoming conceptually more distinct, visually identifiable, for better or more often for worse, as it journeys on to meet a different regime of statistical observation and analysis in the decade that follows.

## Notes

1. Jean-Luc Nancy's examination of listening tentatively draws out its difference from vision as understanding (the expression ‘I see’ captures the elision), asking, ‘Hasn’t philosophy superimposed upon listening, beforehand and of necessity, or else substituted for listening, something else that might be more on the order of *understanding*?’ (2007: 1; original emphasis).

2. A late-life reflection by Birdwhistell on the method that he developed and became known for, of audiovisual sequence analysis, its advantages and dangers, appears in the transcription of a talk at the American Museum of Natural History from 1980. The text was discovered by Seth Watter at the Rare Book and Manuscript Library at Columbia University, mixed in with Jane Howard's papers as part of her research on Margaret Mead (Watter, 2021).

3. The broad understanding of intention as driver of social communication can be seen in the work of literary scholar Kenneth Burke, in a collection of his writings from 1930 to 1960, *Language as Symbolic Action: Essays on Life, Literature and Method*, wherein he distinguishes ‘movement’ as non-symbolic motion, from ‘action’, a symbolically engaged and meaningful gesture (Burke, 1966).

4. The lecture was subsequently published as ‘An Experimental Approach to the Psychopathology of Childhood: Autism’, in the *British Journal of Medical Psychology* (1958a).

5. Anthony's obituary in the American Academy of Child and Adolescent Psychiatry, 11 December 2014, available at: https://www.aacap.org/AACAP/Press/Press_Releases/2014/E_James_Anthony_MD_FRCPsych_1916-2014.aspx.

6. The first experiment with war-traumatized soldiers was closed after only six weeks when the military discovered the nature of the experiment: the men were required to do an hour's exercise a day and to join a group. In Bion's view, the key to rehabilitation was group membership skills, and when the men asked to set up a dance class, he approved (Bion, 2018[1985]).

7. In the lecture Anthony gave to the British Psychological Society as a summary of his clinical research into childhood psychosis.

8. Cinemetric analysis also reveals the film's informational weight with a third of the sequences (88) being intertitle cards, and two-thirds observational recordings (164). In film studies, the scholar Yuri Tsivian has demonstrated the merits of cinemetrics when applied to fiction film, to ‘anatomise a movie’ and lay bare the extent to which the aesthetic foundation of film rests with technical decisions (Tsivian, 2015).

9. Lauretta Bender's use of electroshock therapy on autistic and schizophrenic children at Bellevue Hospital, New York, between 1942 and 1955 in the name of experimental research has been revisited by historians, including in the Autism History Project. See ‘Lauretta Bender, 1897–1987’, Autism History Project, available at: https://blogs.uoregon.edu/autismhistoryproject/people/lauretta-bender-1897-1987/.

10. Home movies would continue to be regarded as evidential documents of child development, evidencing such wide-ranging effects as ‘constitutional qualities of the children, neuromuscular pathology, initial signs of psychosis, and maternal-infant interaction’ (Massie, 1978: 29).

11. It is curious that Anthony would use photographs towards the beginning of both *Aspects of Childhood Psychosis* and *The Natural History of a Psychotic Illness in Childhood*, given that the opening paragraph of Kanner's famous ‘preliminary report’ on autism had signalled photographs of the children had been omitted due to the limitations of space.

12. A filmed case study combining clinical observation with narrative form was made in 1961 by a Dutch psychiatrist, Dr L. N. J. Kamp, who made a series of four detailed clinical films over a period of five years. *Autistic Syndrome*, filmed in a clinical setting in Utrecht, tracks the development of Josie, one of a pair of monozygotic twin sisters. With a running time of approximately 45 minutes each, the episodes document the evolving intimate relationship between the doctor and the child through the use of close, mobile camerawork. The films provide a fascinating example of a clinical case study of childhood autism in which the clinician is invested in narrating events as a life story (Kamp, 1961–6). The films present a rare instance of a child with atypical development being treated to the methods of narration reserved, as Katie Joice has argued, for the typical family child whose life is given story form (Joice, 2022).

13. Henning Engelke discusses the convergence of interest in modernist film-making techniques and psychological research in ‘surface reading’ by avant-garde film-maker Stan Brakhage, who acquired the comprehensive collection of Ray Birdwhistell's writings on body motion interaction. In a lecture, he writes, ‘Brakhage cited Birdwhistell to explain how silent film highlighted and depended on “the particularities of people's motion and speech”, revealing how bodily gestures were interrelated with cinematic technology’ (Engelke, 2021: 130).

14. Seth Watter writes, ‘Kinesics was easily vulgarized, as soon became apparent, and Birdwhistell would long complain of “the popular charlatans of body motion” who made his work seem like a way to pick up women. He himself had designed kinesics in such a rigorous fashion that very few people ever learned its orthography’ (Watter, 2021: 250).

15. For a more detailed account of Birdwhistell's development of a technical language of body movement and the problematic nature of reading context from microanalysis, see Lampert (2019).

16. Wellcome Collection, Sigmund Foulkes personal papers and publications archive.

17. Anthony's obituaries describe a ‘lightness’ to his manner and ‘warm personal friendships’ with figures from different, and in some cases warring, affiliations to psychiatry and psychoanalysis, such as Anna Freud, John Bowlby, Jean Piaget, and Erik Erikson. Unafraid to combine influences from different quarters, he notes Anna Freud's influence on his thinking: ‘I found myself developing another close relationship with Miss Freud that enhanced my ideas of early development’ (Anthony, 2010).

18. In Henrik Engelke's account of the NHI, what is overlooked by the researchers, surprisingly and uncomfortably given the gender dynamic, was the participant's reservations: ‘Rather than getting used to being filmed, Doris’ discomfort grew’, he writes (Engelke, 2021: 106).

19. Autistic rhetorician Remi Yergeau argues that intention has operated as a key diagnostic marker of deficit in the history of autism, suggesting, in place of this invidious concept, the term ‘involution’, a hybrid of intention and revolution (Yergeau, 2018).
